# Long-Term Survival and PSA Control with Radiation and Immunotherapy for Node Positive Prostate Cancer

**DOI:** 10.1155/2009/363914

**Published:** 2010-03-08

**Authors:** M. Kamrava, Kwong Y. Tsang, R. A. Madan, A. Kaushal, C. N. Coleman, J. Gulley

**Affiliations:** ^1^Radiation Oncology Branch, Center for Cancer Research, National Cancer Institute, Bethesda, MD 20892, USA; ^2^Laboratory of Tumor Immunology and Biology, Center for Cancer Research, National Cancer Institute, Bethesda, MD 20892, USA

## Abstract

We describe a patient with node positive prostate cancer treated with radiation, androgen deprivation, and immunotherapy with long-term overall survival and PSA control. ELISPOT immunoassay studies demonstrated PSA specific T-cells prior to starting vaccine therapy suggesting that this positive response may be related to an improved antitumor immune response of the patient, increased immunogenicity of the tumor, or decreased activation of immune escape pathways. Further evaluation of therapeutic cancer vaccines in combination with radiation and hormonal therapy in the definitive management of prostate cancer is warranted.

## 1. Introduction

Men with newly diagnosed node positive prostate cancer have a higher likelihood of developing progressive disease and dying from prostate cancer than men without lymph node involvement [1]. The current standard of care for these men includes a combined modality approach with radiation and androgen deprivation therapy to address both locoregional and systemic disease. Results with this approach are not ideal and the addition of other modalities, such as immunotherapy, to radiation and hormonal treatment may improve treatment outcomes [2].

We present a case report on a node positive prostate cancer patient treated with immunotherapy, radiation, and androgen deprivation with long-term overall survival and PSA control.

**Case 1**

**Figure 1:**
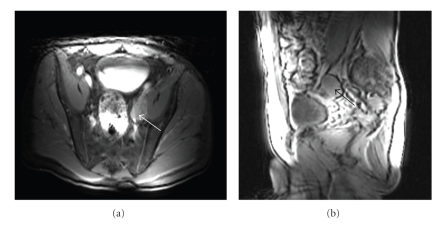
Axial and sagittal magnetic resonance images showing an enlarged left external iliac lymph node (white arrow axial, black arrow sagittal).

This patient is a 59-year-old male who initially presented with signs and symptoms of prostatitis. PSA ordered as part of his work-up was 95 *μ*g/L. He was treated with 2 courses of antibiotics, after which a repeat PSA was 95 *μ*g/L. A subsequent transrectal ultrasound-guided biopsy revealed adenocarcinoma of the prostate in 7/8 cores, with Gleason score 4 + 3 being the highest grade seen. Further evaluation included a computed tomography scan of the abdomen and pelvis, magnetic resonance imaging of the pelvis showing a normal-sized prostate with no evidence of extracapsular extension, a periprostatic lymph node measuring 1.8 cm, a left iliac lymph node measuring 2.5 × 3.8 × 3.8 cm (Figure 1), and a normal bone scan. Digital rectal examination demonstrated an abnormality in more than half of one lobe but not both. The remainder of his physical examination, laboratory tests, and history was unremarkable. The patient was clinically staged as Stage IV (T2bN1M0).

**Figure 2:**
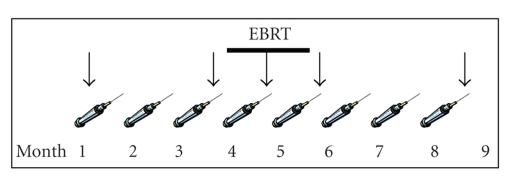
Three monthly vaccines were given before external beam radiation therapy (EBRT, thick line). The initial vaccine was given with an admixture of rV-PSA and rV-B7.1 with follow-up monthly boosts given with recombinant fowlpox PSA. Immunologic studies were performed prevaccination, post-3, post-4, post-5, and post-8 cycles of vaccine (designated by the arrows).

The patient was subsequently enrolled on a randomized phase 2 clinical trial at the National Cancer Institute, where he was treated with a priming vaccine of recombinant vaccinia virus engineered to encode PSA admixed with a recombinant vaccinia virus encoding the T-cell costimulatory molecule B7.1. This was followed by monthly boosts with a recombinant fowlpox vector expressing PSA, for a total of 8 vaccinations [3] (Figure 2). The vaccines were given with local granulocyte-macrophage colony-stimulating factor and systemic low-dose interleukin-2 (4 MIU/M^2^).

External beam radiation therapy was given between the fourth and sixth vaccinations. The patient was treated using 3D conformal radiation therapy to a dose of 75.6 Gy, with the left iliac lymph node receiving 59.4 Gy. He was started on bicalutamide and goserelin prior to starting radiation therapy and continues on this regimen. 

At his last follow-up, 98-month postradiation treatment, the patient's PSA was <0.04 *μ*g/L. 

## 2. Methods

Correlative immunologic studies were performed that measured PSA-specific T cells with ELISPOT prevaccination, post-3, post-4, post-5, and post-8 cycles of vaccine as previously described [2].

## 3. Results and Discussion

Mature biochemical progression data from the trial the patient in this case study was enrolled on are not yet available. Among the 8 patients with node-positive disease enrolled on the study however, 5 have developed biochemical progression at 1, 7, 58, and 68 months. We do not have the exact progression date for the fifth patient but know that he progressed within 48 months from the end of his radiation treatment. The other 2 patients have not had biochemical progression as of follow-up at 63 and 71 months. Immunologic studies were performed on 4 of the node-positive patients, and the patient presented here is the only one of the 4 who has not developed biochemical progression. Interestingly, he is the only patient among this group to have PSA-specific T cells (i.e., >1: 100,000) prior to starting vaccine and the most vigorous T-cell response post vaccination (Table 1).

This may suggest that, compared to others in the group, this patient's tumor may have had more HLA class I and II molecules loaded with relevant prostate cancer-associated antigens making immune detection of the tumor easier (more immunogenic), there was more upregulation of fas on the tumor (making it easier to kill immunologically), or there was decreased negative immune regulation. Interestingly, his level of Treg cells (CD4^+^CD25^high^Foxp3^+^) was not elevated (2.1% of CD4 cells) [5]. Furthermore, additional testing showed that his PSA-specific T-cell response remained detectable (between 1/13,953 and 1/86,714) at each time point tested (3, 6, 12, and 24 months) following completion of vaccine. It is also possible that by boosting an already present relevant antitumor immune response, he may have benefited more from immunotherapy than the other patients without a baseline antitumor immune response. 

While this hypothesis is intriguing, these 4 patients did have different baseline characteristics (Table 2). 

This patient's PSA was higher than the group median of 41 *μ*g/L; however, he received a slightly higher dose of radiation compared to the median 72.9 Gy and was the only patient who received a radiation boost to his involved lymph node. He also had a Gleason score of 7, which was lower than the scores of 8 to 9 for the other 3 patients. In addition, he has been on androgen deprivation with bicalutamide and goserelin since his diagnosis (about 7.5 years) whereas the longest time of hormone treatment, with either goserelin alone or goserelin and bicalutamide, for the other three patients was 2 years. These differences preclude drawing any definite conclusions regarding whether this patient truly benefited from vaccine therapy. However, it is noteworthy that the only node-positive patient with PSA-reactive T cells prior to starting vaccine is the only one who is still PSA failure-free at 98 months. 

There are limited long-term data on patients with node-positive prostate cancer treated with radiation therapy. The Radiation Therapy Oncology Group (RTOG) 75-06 study treated patients with node-positive prostate cancer with radiation therapy to either a pelvic or pelvic plus para-aortic field. At 10 years, 5/90 patients were alive and clinically free of cancer without hormone therapy [6]. In a follow-up study, RTOG 85-31 randomized men to immediate androgen suppression with radiation versus radiation alone and hormonal therapy started at time of relapse. This study included a subset of 173 node-positive prostate cancer patients. Within this group, those who received immediate hormone therapy were more likely to have a PSA <1.5 ng/mL at 9 years versus those who received delayed hormone therapy (10% versus 4%, *P* < .0001) [7]. While adding hormones to radiation therapy certainly improves outcomes, there is much room for improvement. 

Immunotherapy may be a promising option for achieving this outcome. Results from IMPACT (Immunotherapy for Prostate AdenoCarcinoma Treatment), a randomized phase 3 study of Provenge (sipuleucel-T) versus placebo in metastatic androgen-independent prostate cancer, showed a significant survival benefit of 4.1-month median overall survival for patients receiving Provenge [8]. Furthermore, a multicenter-randomized controlled phase II study of PROSTVAC (a similar vaccine to the one described here but with additional costimulatory molecules) demonstrated an 8.5-month improvement in median overall survival, the secondary endpoint of the study, in patients with metastatic castration resistant prostate cancer [9]. There is also a suggestion that patients treated with lower volumes of prostate cancer may have a larger survival benefit with immunotherapy [10]. Whether immunotherapy plus radiation and hormonal therapy for men with localized or locally advanced prostate cancer offers an advantage to standard treatment alone is currently unknown.

The patient in this case report had reactive T-cells prior to vaccine administration and is the only one of the node positive patients on trial who is still PSA failure free. While it is not proved clearly, this suggests that patients with intact immune surveillance mechanisms may be more likely to respond to treatment with immunotherapy.

## 4. Conclusions

Given the recent survival benefit seen with immunotherapy in metastatic prostate cancer patients, the role of immunotherapy in a definitive setting with standard treatments merits further investigation.

## Figures and Tables

**Table 1:** ELISPOT PSA-specific T cells for patients with node positive disease at various time points during vaccination. Patient 1 is the only patient with detectable PSA-specific T cells prior to starting vaccination. It should be noted that the post-4 immune responses were obtained mid-radiation, and unlike the other time points, this blood was prepared from a peripheral blood draw rather than an apheresis sample. Interestingly, radiation has been shown to upregulate markers on the tumor facilitating tumor specific T-cell trafficking which may impact peripheral circulation of antigen specific T-cells (reviewed in [4]). It is possible that either of these variables could have impacted the measured immune response. *Post-2 cycles.

Time Point	Patient 1	Patient 2	Patient 3	Patient 4
Pre-vaccine	1/50,000	1/200,000	<1/200,000	1/150,000
Post-3 cycles	1/37,500	1/54,545	1/50,000	<1/200,000*
Post-4 cycles	<1/200,000	<1/200,000	<1/200,000	Data not available
Post-5 cycles	1/24,000	1/66,667	<1/200,000	1/12,000
Post-8 cycles	1/15,789	1/22,222	1/46,154	Data not available

**Table 2:** Baseline patient characteristics and clinical/PSA failure status for patients with immunologic data and node positive disease.

Patient #	Age at diagnosis (years)	Clinical stage	Gleason score	PSA at diagnosis (*μ*g/L)	Total dose radiation (Gy)	Clinical/PSA failure
1	51	T2bN1M0	7	95	75.6	No
2	56	T2cN1M0	9	5	70.2	Yes
3	56	T2cN1M0	8	63	68.0	Yes
4	55	T2aN1M0	8	19	75.6	Yes
